# Wafer-Scale Fabrication and Transfer of Porous Silicon Films as Flexible Nanomaterials for Sensing Application

**DOI:** 10.3390/nano12071191

**Published:** 2022-04-02

**Authors:** Han Lu, Mingliang Jin, Zongbao Zhang, Sujuan Wu, Lingling Shui

**Affiliations:** 1 School of Information and Optoelectronic Science and Engineering, South China Normal University, Guangzhou 510006, China; hanlu@m.scnu.edu.cn; 2 National Center for International Research on Green Optoelectronics, South China Academy of Advanced Optoelectronics, South China Normal University, Guangzhou 510006, China; 3 International Academy of Optoelectronics at Zhaoqing, South China Normal University, Zhaoqing 526238, China; 4 Institute for Advanced Materials, South China Academy of Advanced Optoelectronics, South China Normal University, Guangzhou 510006, China; zongbao.zhang@iapp.de (Z.Z.); sujwu@scnu.edu.cn (S.W.)

**Keywords:** surface-enhanced Raman scattering, flexible sensor, porous silicon film, large-area fabrication

## Abstract

Flexible sensors are highly advantageous for integration in portable and wearable devices. In this work, we propose and validate a simple strategy to achieve whole wafer-size flexible SERS substrate via a one-step metal-assisted chemical etching (MACE). A pre-patterning Si wafer allows for PSi structures to form in tens of microns areas, and thus enables easy detachment of PSi film pieces from bulk Si substrates. The morphology, porosity, and pore size of PS films can be precisely controlled by varying the etchant concentration, which shows obvious effects on film integrity and wettability. The cracks and self-peeling of Psi films can be achieved by the drying conditions after MACE, enabling transfer of Psi films from Si wafer to any substrates, while maintaining their original properties and vertical alignment. After coating with a thin layer of silver (Ag), the rigid and flexible PSi films before and after transfer both show obvious surface-enhanced Raman scattering (SERS) effect. Moreover, flexible PSi films SERS substrates have been demonstrated with high sensitivity (down to 2.6 × 10^−9^ g/cm^2^) for detection of methyl parathion (MPT) residues on a curved apple surface. Such a method provides us with quick and high throughput fabrication of nanostructured materials for sensing, catalysis, and electro-optical applications.

## 1. Introduction

Porous silicon (PSi) has attracted much attention due to its potential applications in energy storage, drug delivery, and biosensors [1,2,3,4,5]. Pore size and porosity of PSi strongly influence its mechanical, optical, and electrical properties, thus determining its performances in practical applications [6]. Up until now, three methods have been developed to fabricate PSi, which include stain etching, electrochemical etching, and metal-assisted chemical etching (MACE) [7,8,9]. Stain etching has a limited etching depth and lacks the controllability of pore size and porosity. Electrochemical etching permits to control over the pore size and porosity via tuning the applied current, as well as the HF electrolyte concentration. However, electrochemical etching requests corresponding chemicals and electrical equipment.

Recently, MACE has often been used to fabricate PSi [10,11] due to its low cost and simplicity. This technique has been proposed to produce large-area silicon nanowire arrays [12]. MACE is a purely solution-based and high-throughput technique, mainly including two steps, the nucleation of metal nanoparticles, and the anisotropic etching in a solution containing HF and oxidant agents like H_2_O_2_ [13]. However, the formed PSi attached in Si substrate limits their application flexibility, making it difficult to manipulate its physical shapes. Mechanical flexibility can bring an important degree of freedom in designing, while maintaining a high performance. Flexible PSi films are much more convenient to conform to surfaces with various curvatures and shapes. Detaching PSi from parent Si wafers allows to isolate and harness PSi’s electrical, thermal, optical, and mechanical properties in devices without being overshadowed by the properties of the thick parent Si wafers; meanwhile, at the same time, enables the transfer of required PSi to other flexible, lightweight, low cost, or transparent substrates for enhanced device functionality. One of the key requirements for the separation and transfer of PSi is to preserve their original properties and orientation, especially for transferring to flexible substrates by maintaining their vertical structures.

There have been many reports on transferring various types of Si nanostructures (such as nanowires and PSi) onto paper, plastic, polymer, or metal substrates, especially for applications in the fields of surface-enhanced Raman scattering (SERS), lithium-ion batteries, and drug delivery [13,14,15,16]. Wang et al. obtained SiNWs embedded poly(vinyl alcohol) (PVA) films by spin-coating and mechanically peeling PVA with SiNWs [16]. Weisse et al. transferred cracked vertical SiNW arrays into poly(methyl methacrylate) (PMMA) by inserting a water soaking step between two consecutive Ag-assisted chemical etching steps [17], and SiNW arrays to a flexible stainless-steel sheet by electro assisted transfer using a sacrificial porous silicon layer [18]. Wang et al. also achieved transferring SiNWs onto flexible substrates by preparing NWs using an air heating approach and transferring through a roll-to-roll technique [19]. However, these methods rely on the mechanical breaking or multiple chemical steps and are only suitable for small-size substrates.

In this work, we fabricated wafer-scale PSi film on silicon wafer using MACE. Via controlling over horizontal cracks in the drying process, flexible PSi films can be obtained by simply pasting and peeling an adhesive tape from the wafer surface. The influence of etchant concentration on the morphology, porosity, and pore size of the formed PSi films are systematically investigated. After coating a thin layer of Ag film, the flexible PSi films show promising SERS performance for highly sensitive molecular detection. This opens opportunities for wider applications of PSi films in solar cells, SERS, biosensors, as well as fundamental studies on their physical properties.

## 2. Materials and Methods

### 2.1. Materials

Hydrofluoric acid (HF, 49 wt%) and potassium hydroxide (KOH, GR 95%) were purchased from Aladdin (Shanghai, China). Hydrogen peroxide (H_2_O_2_, 30 wt%), sulfuric acid (H_2_SO_4_, 98 wt%), and ethanol were of analytical grade and received from Guangzhou Chemical Reagent Factory (Guangzhou, China). Photoresist SUN-120P was purchased from Suntific Microelectronic Materials Co., Ltd. (Weifang, Shandong, China) for patterning Si. 4-Methylbenzenethiol (4-MBT, 98%) was purchased from Sigma-Aldrich (St. Louis, MO, USA). Rhodamine 6G (R6G) was purchased from J&K Chemical (Beijing, China). Methyl parathion solution (MPT, C_8_H_10_NO_5_PS, 100 μg/mL in methanol) was obtained from Sinopharm Chemical Reagent Co., Ltd. (Shanghai, China). DP-type (100) 4” Si wafer with a resistivity of 0.001–0.005 Ω·cm was purchased from Lijing Optoelectronics Co., Ltd. (Suzhou, China). Deionized (DI) water (18.25 MΩ·cm at 25 °C) was prepared using a Milli-Q Plus water purification system (Sichuan Wortel Water Treatment Equipment Co., Ltd., Chengdu, Sichuan, China). All chemicals were used as received.

### 2.2. Fabrication of PSi Films

Si wafers were used as the substrates for surface patterning and MACE to prepare PSi structures. A Si wafer was first ultrasonically cleaned in DI water for 15 min, then immersed in Piranha solution for 15 min and thoroughly rinsed using DI water. Photoresist SUN-120P was spin coated on the cleaned Si surface using a Smart Coater 100 (Best Tools, LLC, St. Louis, MO, USA) at 3000 rpm for 60s. After blow drying using N_2_ gas, the wafer was put on to the stage of an aligner (URE-200/35, Institute of Optics and Electronics, Chengdu, China), being exposed for 30s under UV light with an intensity of 13 mW/cm^2^. Developing of the photoresist was carried out in 0.5 wt% KOH at 25 °C for 2 min. Patterned photoresist was then obtained by rinsing using DI water, drying using a nitrogen gun, and then hard baking on a hot plate (EH20B, Lab Tech, Beijing, China) at 120 °C for 30 min. Afterward, a 120 nm thick Ag was deposited onto the patterned Si wafer using thermal evaporation (BM450D, Ke You Vacuum Technologies Co., Ltd., Shenyang, China) at a deposition rate of 0.5 A·s^−1^. Subsequently, the lift-off of photoresist was done by sonicating in ethanol at room temperature for 2 min. Obtained substrate was then immersed and etched in the etchant solution, a mixture of HF, H_2_O_2_, and DI water, in a Teflon container. Afterwards, the MACE sample was taken out, rinsed using DI water, and dried using a nitrogen gun to obtain PSi films on a Si wafer. The horizontal cracks and self-peeling formed during drying after MACE step.

### 2.3. Vertical Transfer PSi Films to Flexible Substrate

An adhesive tape was pasted on the top of a PSi film and pressed with uniform stress to ensure it tightly adhered on the surface. Afterward, the tape was slowly peeled off from the Si substrate with the PSi adhered to the adhesive, being ready to be transferred to any substrate.

### 2.4. Wettability Measurements

The surface wettability was measured using OCS 15pro (Dataphysics, Stuttgart, Germany). Each sample was measured three times at different locations by using a DI water droplet of about 2 μL. The contact angle (CA) of as-prepared PSi films, with HF treatment (10 wt% HF for 1 min), and after transfer onto flexible tape was measured.

### 2.5. SERS Measurements

PSi film consists of a large number of pores. When Ag was deposited onto the surface, the metal covered nano-gaps (nanopores) serve as hotspots to reach surface plasmonic effect. The substrate was immersed in the analyte solution for 1 h, and then thoroughly rinsed for 1 min using the corresponding solvent and blow dried with pure nitrogen gas. The dried substrate was then measured by placing on the stage of a Renishaw inVia Raman Microscope (Renishaw 42K846, Renishaw Co., Ltd., Gloucestershire, UK) with an excitation laser of 532 nm and power of ~0.14 mW. The laser beam was focused on the sample through a 50× objective lens (NA = 0.5, Leica). The diameter of the laser spot was 1.30 μm. The elastically scattered laser excitation was removed with an edge filter. Each Raman spectrum was collected with accumulation time of 10s.

### 2.6. Other Characterizations

The morphologies of PSi films were characterized using a desktop scanning electron microscopy (SEM) (Phenom G2 Pro, Phenom-World, Eindhoven, The Netherlands) and a Field Emission-SEM (FE-SEM) (ZEISS-Ultra55, Carl Zeiss AG, Oberkochen, Germany). The reflection spectra of the PSi films before and after transferred onto the tape were both recorded in the wavelength range of 450–700 nm by using a spectrometer (USB 2000+, Ocean Optics, Dunedin, FL, USA) and UV-VIS-NIR spectrophotometer (Lamda 750s, PerkinElmer Inc., Shelton, CT, USA).

## 3. Results

### 3.1. Fabrication of PSi Films on Si Substrate

**Figure 1:**
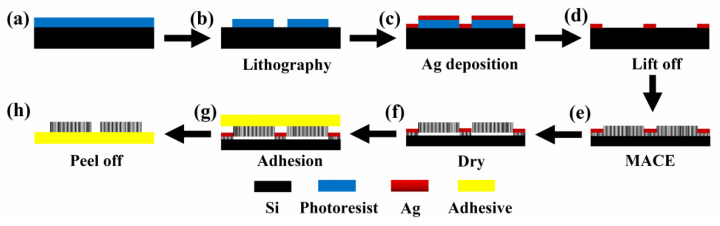
Schematic of the transfer process of vertical PSi film. (**a**) Spin coating of photoresist, (**b**) lithography, (**c**) Ag deposition, (**d**) lift off, (**e**) MACE, (**f**) drying, (**g**) adhesion, (**h**) peel off.

Figure 1 illustrates the step-by-step fabrication and transferring of a PSi film. Patterned Si wafer is first patterned via a standard photolithography process (Figure 1b). The Ag film is then deposited on the patterned Si surface to serve as catalyst for the following MACE process (Figure 1c). Subsequently, the lift-off of photoresist is carried out by sonicating in ethanol (Figure 1d). PSi film containing closely arranged nanopores is then obtained according to MACE by immersing the substrate in the etchant solution (Figure 1e). Horizontal cracks are subsequently produced during the PSi film drying process, leading to the self-peeling of formed PSi film (Figure 1f). Finally, the PSi film is easily detached from the Si substrate using an adhesive tape, being ready to be used or attached to any surfaces (Figure 1h).

PSi is formed on Si wafer surface without metal coverage as observed in previous work [20,21]. During the etching process, Ag can be oxidized to Ag^+^ by H_2_O_2_. Alternatively, these Ag^+^ can be quickly reduced back to Ag at the interface of Ag and Si. Pores were formed by attacking from the redeposited Ag nuclei [13,21,22], as described in Equations (1) and (2) [20], and schematically shown in Figure S1. Formed nanopores are highly uniform (as shown in Figure S2), with the etching rate is nearly the same as the MACE rate below Ag films.

(1) $$2\mathrm{Ag}+H_{2}O_{2}+2H^{+}\to 2{\mathrm{Ag}}^{+}+2H_{2}O$$

(2) $$\mathrm{Si}+4{\mathrm{Ag}}^{+}+6F^{-}\to 4\mathrm{Ag}+{\mathrm{SiF}}_{6}^{2-}$$

**Figure 2:**
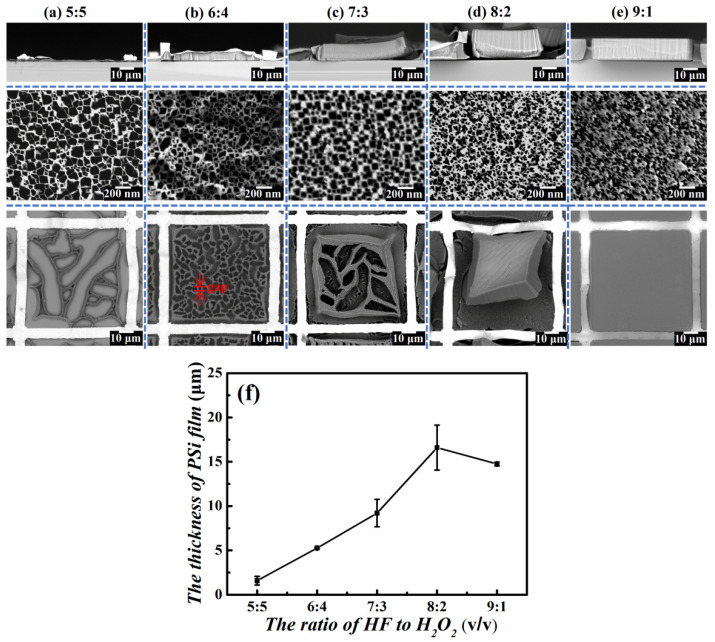
SEM images (top: cross-sectional view, middle: high resolution of top view, bottom: top view) of a patterned Si etched in different *R* (**a**) 5:5, (**b**) 6:4, (**c**) 7:3, (**d**) 8:2, and (**e**) 9:1 for 30 min, respectively. (**f**) The thickness of PSi film as a function of the *R*.

The pore size and porosity of PSi are dependent on the etchant composition, mainly the ratio of HF to H_2_O_2_ [23,24]. To investigate this factor, five volume ratios of HF to H_2_O_2_ (*R*) were applied, which were 5:5, 6:4, 7:3, 8:2, and 9:1. The substrates were patterned to 65 × 65 μm^2^ squares covered with ~120 nm Ag, being separated by 5 μm gaps. As shown in Figure 2, PSi films formed on the top surface of Si wafers, with various pore size, film thickness, and cracks. The average pore size and porosity of ~89 nm and ~74% (Figure 2a), ~72 nm and ~68% (Figure 2b), ~53 nm and ~34% (Figure 2c), ~30 nm and ~25% (Figure 2d), and ~22 nm and ~16% (Figure 2e), were obtained by reaction for 30 min in the etchants with *R* of 5:5, 6:4, 7:3, 8:2, and 9:1, respectively. The corresponding pore size distribution histograms of PSi films were presented in Figure S3. PSi forms according to the attacking from the redeposited Ag nuclei. The dissolution and nucleation of Ag is a highly dynamic process and closely related to the Si etching process. Increasing the ratio of HF to H_2_O_2_ leads to a decreased number of holes available for injection into Si, thus the porosity is decreased. The pore size is strongly depended on the ratio of HF to H_2_O_2_ [13,25]. The Ag^+^ preferentially nuclei around generated Ag nanoparticles, rather than exposed Si, and eventually forming larger Ag particles [24]. The etching rate is dependent on the ratio of HF to H_2_O_2_. A higher *R* will lead to difficultly form lager particles. As a result, the size of formed pores decreased with the increase of *R*. The thickness of the PSi films was measured by using a SEM, and Figure 2f presents the plot of the obtained film thickness corresponding to *R* of the etchant.

To our surprise, although the film thickness increases and the pore size decreases from Figure 2a–d, the cracks on the same sized film decrease. As we know, thicker films are easy to crack, and a smaller pore size would induce higher capillary pressure during drying. Therefore, the difference of cracks is mainly attributed to the decrease in porosity. The PSi film cracked to long “lines” (Figure 2a, 74% porosity), small connected “pieces” (Figure 2b, 68% porosity), or big connected “pieces” (Figure 2c, 34% porosity). When the porosity decreased to about 25% (Figure 2d), a complete piece of PSi film formed with some crevices along the bending longitude of the curved surfaces (this might be caused by the bending of the surface). In Figure 2e, although the films were ~15.1 μm thick, the whole pieces of PSi films kept their integrity after drying. This result is consistent with previous reports [25,26].

### 3.2. Transfer of PSi Films

**Figure 3:**
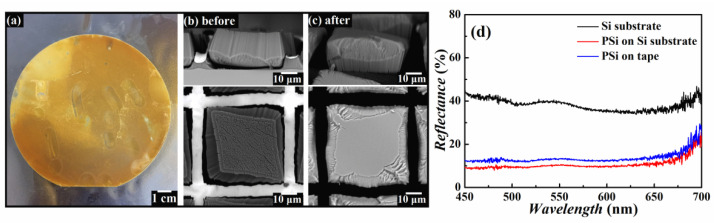
(**a**) Optical image of a large area (4”) transferred PSi films onto flexible tape. SEM images (top: cross-sectional view, bottom: top view) of PSi films (**b**) before and (**c**) after transfer. (**d**) Reflectance spectra of PSi films before and after transfer onto tape.

As described above, PSi films form cracks horizontally and self-peeling from the Si substrate vertically as shown in Figure 2. As a result, these films can be easily detached from Si surface. Vertical transfer of the PSi films was then achieved by attaching and detaching a large adhesive tape as a receiver substrate on the top of PSi films, as drawn in Figure 1g. When the adhesive tape was peeled from the Si substrate, PSi films were taken away together with it. Figure 3a shows image of a tape covered with PSi substrate transferred from a 4-inch Si wafer prepared via MACE in an etchant with *R* = 8:2 for 30 min. The sample patterns were 50 × 50 μm^2^ square with gap distance of 5 μm. Figure 3b,c shows the SEM images of a PSi film before and after transferring onto a flexible tape, indicating the successful transferring process has been achieved with vertical pores retained on a large-area flexible surface. This is particularly important for their practical applications, since most of the distinct properties of the PSi are related to their high porosity and vertical alignment. Figure 3b shows a vertically peeled PSi film with uniform thickness (~20.8 μm) and horizontal crevices. After being transferred to the tape, the pores were kept vertical to the tape surface, as shown in Figure 3c corresponding to the drawing in Figure 1h. The transfer process was very thorough and does not make obvious damage to the whole PSi films. Almost all PSi films on the Si wafer were transferred to the flexible tape.

Figure 3d shows the reflectance spectra of the PSi films before and after transfer on the tape. Notably, the reflectance of the transferred PSi films is much lower than the flat Si substrate. The low reflectance of the PSi films is attributed to the light trapping effect of nanopores, which can effectively suppress the reflection on the Si wafer surfaces, in agreement with previous work [27]. The reflectance spectra of the PSi films are comparable to the PSi films on Si substrate. This result clearly demonstrates that the transferred PSi films is kept and vertical to the tape surface and can still maintain the excellent anti-light-reflection property.

The key factor of this transfer method is the formation of horizontal cracks and self-peeling of PSi pieces during the drying process. The dominant factor is the capillary force, which is responsible for high tensile stresses during drying, and leads to the destruction of the PSi film from Si substrate. The mechanism of crack formation and self-peeling during drying has been reported previously [25,26,28,29,30,31,32]. During drying, when liquid evaporates from the pores, the PSi films are under lateral stress and stress normal to surface and will shrink or crack to minimize the total surface and reduce the total energy of the system. When the stress exceeds a critical limit, physical separation is obtained between the PSi layer and the bulk Si substrate, schematically described in Figure S4.

### 3.3. Surface Wettability

**Figure 4:**
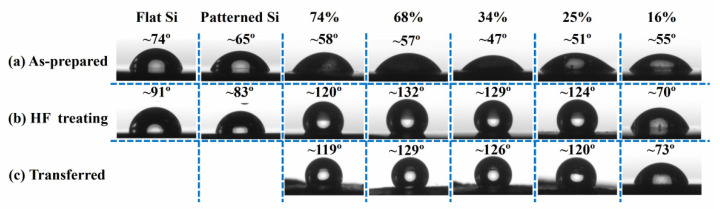
Water CAs on flat Si, patterned Si, and PSi films (**a**) as prepared, (**b**) after HF etching, and (**c**) after transfer onto a tape. The porosity is marked on top of each image.

The surface wettability of as-prepared PSi films before and after transfer was investigated by measuring the water contact angle (CA). Figure 4 shows the apparent water droplet CAs on various surfaces, including flat Si substrates, patterned Si substrates covered with Ag film, as-prepared PSi films, PSi films with HF treatment, and PSi films after transfer. These films were prepared under the same conditions as those of Figure 2a–e, respectively, and thus showing porosity in the range of 74–16%.

The flat Si and patterned Si substrates are hydrophilic with CAs of ~70°. And the PSi films show small CA of 47–58°, regardless of porosity and morphologies, as shown in Figure 4a. This is due to the formation of an oxide layer on PSi surface during MACE process. Whereas, after HF etching, the PSi films (porosity of 74–25%) exhibit large CA of ~130°, as shown in Figure 4b. The wettability of solid substrates is governed by surface free energy and geometrical structure [33]. The significant CA change from hydrophilic to hydrophobic is due to the removing of the oxide layer to explore the bare silicon surface. The co-existence of nanopores and micro-gaps can trap air in the pores and gaps to support water droplets, leading to high CAs. When the porosity decreases to ~16%, CA reduces to ~70°, being similar to bare Si surface. This is not surprising, since they might be due to the decrease of the contribution from the nanopores, which cannot trap air any longer. Figure 4c presents the CAs on PS films after transferred to the tape, showing similar CAs as those on the prepared PSi structures on Si substrate before transferring. This suggests the success of the transferring process without damage of the microstructures.

### 3.4. SERS Measurements

**Figure 5:**
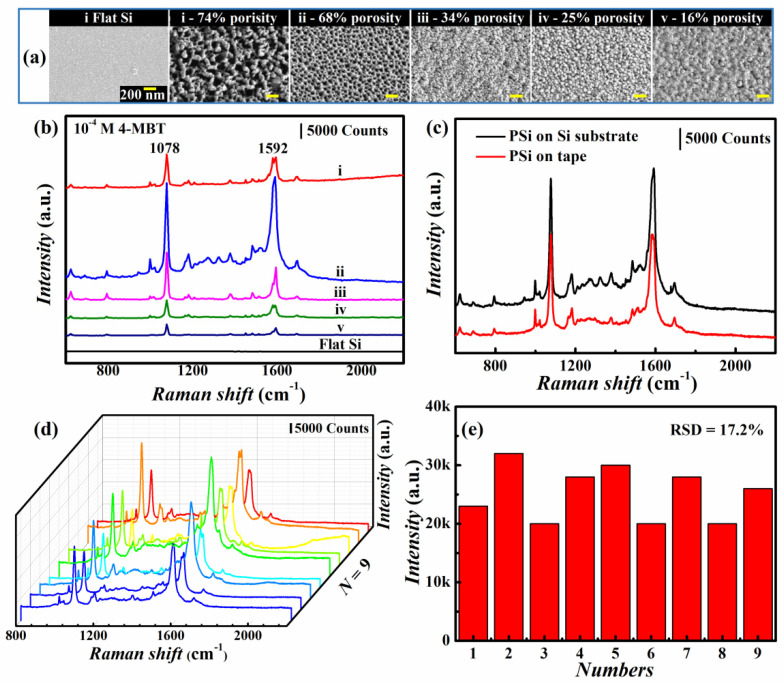
(**a**) SEM images of the samples for SERS characterization. (**b**) Raman spectra of 10^−4^ M 4-MBT collected on the samples of (**a**). (**c**) Raman spectra of 10^−4^ M 4-MBT collected on the optimized PSi films (sample ii) before and after transfer. (**d**) Raman spectra of 10^−4^ M 4-MBT from nine different PSi films substrates under optimized parameters corresponding to sample ii. (**e**) Raman intensity distribution of the peak 1078 cm^−1^ of the nine Raman spectra from (**d**).

Nanopores possesses advantages for light manipulation and sensing applications [34,35,36]. Hereby, we demonstrate the PSi films as excellent candidates for SERS application due to the advantages of large specific surface area, environmentally friendly, biocompatibility, and easy and high throughput preparation and patterning. Moreover, in comparison to rigid SERS substrates, the flexible PSi films on tape had the advantages of being lightweight and easy to handle. The chosen probe molecule was 4-MBT, and the PSi films before and after transferred on tape coated with 40 nm Ag were used as the SERS substrate. The reference substrate was prepared by directly sputtering 40 nm Ag film on bare flat Si substrate. Figure 5a shows the SEM images of Ag coated substrates. The pores became clogged when pore size decreased to ~53 nm (25% porosity) and formed a continuous film when pore size reduced to ~22 nm (16% porosity).

The Raman spectra of 10^−4^ M 4-MBT collected from the substrates are shown in Figure 5b. The two dominant peaks at 1078 and 1592 cm^−1^ were the characteristic peaks for 4-MBT. The 1078 cm^−1^ was due to a combination of phenyl ring-breathing modes with a C-S stretching mode. The 1592 cm^−1^ peak represented the C-C ring symmetric stretching mode [37]. The intensity of Raman peak of 1078 cm^−1^ increased from ~11,000 to ~28,000 when the porosity and pore size changed from ~89 nm (74% porosity) and ~72 nm (68% porosity). Afterwards, the Raman intensity decreased with the decreasing porosity and pore size, and the pores were fulfilled with Ag and formed continuous film in sample v. The maximum Raman intensity was therefore achieved on the substrate ii. To quantitatively characterize the SERS effect of prepared substrates, we calculated the enhancement factor (EF). The Raman spectra of pure powders of 4-MBT (Figure S5), and the detailed information of EF calculation are presented in the supplementary information. The EFs of the SERS substrates (samples i–v in Figure 5a) were calculated to be ~1.12 × 10^7^, ~2.85 × 10^7^, ~1.68 × 10^7^, ~0.56 × 10^7^, and ~0.27 × 10^7^ at the Raman peak of 1078 cm^−1^, respectively.

Figure 5c shows the Raman spectra collected from the PSi films before and after transferred to tape, showing nearly identical Raman peaks and intensity. The SEM images of PSi films before and after transfer to tape coated with 40 nm Ag film were presented in Figure S6, indicating that the transfer process was successful without affecting nanopore structures, and thus the SERS enhancement. To test whether the flexible PSi film substrate was able to produce repeatable SERS signals, we collected Raman spectra of 10^−4^ M 4-MBT from 9 PSi film substrates optimized under the same conditions corresponding to sample ii in Figure 5a, as shown in Figure 5d. Repeatable results with relative stable Raman intensity have been achieved with and the relative standard deviation (RSD) of ~17.2% at the characteristic 1078 cm^−1^ peak (Figure 5e), revealing the reliability of this SERS sensor. Moreover, R6G had also been selected to demonstrate the SERS substrate’s sensitivity, as shown in Figure S7. The detection limit was measured to be 10^−12^ M, demonstrating the reliable SERS effect.

Flexible substrates attracted much attention in SERS due to the advantage in tunable plasmonic resonances and directly collecting analyte from the curved surface [38,39,40,41,42,43]. As a further proof of the flexibility of our fabricated SERS substrate, the flexible SERS substrates were used for detection of pesticide residues on apple surface with a real curved surface. The minimum detectable concentration of MPT was 10^−6^ M (Figure S8), converted to mass-to-area ratio was 2.6 × 10^−9^ g/cm^2^, which was lower than the maximum residue limit for MPT in China and European Union [44]. The mechanically robust of the flexible PSi films SERS substrate was evaluated by applying bending for 100 cycles. The SERS substrates could still maintain mechanical stability even after 100 cycles of bending (Figure S9).

## 4. Conclusions

A simple and reliable method has been demonstrated for vertical transfer of large-area PSi films with uniform thickness onto flexible substrates in one-step MACE. The morphology, porosity and pore size of PSi films can easily be controlled by tuning the etchant composition. According to the pre-patterned segments, horizontal crack, and detachment of PSi areas from bulk, the Si substrate is achieved to enable direct film transfer to a flexible tape via a simple pasting and peeling process. Transferred PSi films maintained their original nanopore size and vertical alignment. The film integrity and surface wettability of the PSi substrate is mainly determined by its pore porosity. Prepared PSi on Si substrate and transferred on tape have been demonstrated for rigid and flexible SERS substrates, showing high enhancement at the optimized conditions. Such a technology is simple, fast, and flexible for high-throughput production of large-area patterned PSi films. The PSi on tape can be easily handled and integrated into portable Raman spectroscopes for point-of-care diagnostics and biosensors for on-site quick inspection.

## Data Availability

The data presented in this study are available on request from the corresponding author upon reasonable request.

## Supplementary Materials

The following supporting information can be downloaded at: https://www.mdpi.com/article/10.3390/nano12071191/s1, Figure S1: Schematic of PSi by metal-assisted chemical etching; Figure S2: (a) SEM image of PSi films (a 65 × 65 μm^2^ patterned substrate covered with ~120 nm Ag reaction in the etchant with *R* = 6:4 for 30 min). (b) Corresponding distribution histogram of pore size; Figure S3: Corresponding pore size distribution histograms of PSi films with different porosity (a) 74%, (b) 68%, (c) 34%, (d) 25%, and (e) 16% (corresponding to Figure 2a–e); Figure S4: (a,b) Model of the formation of horizontal cracks of PSi films during drying. (c) SEM image of PSi films with horizontal cracks; Figure S5: (a) Raman spectra of 10^−4^ M 4-MBT on PSi SERS substrates, (b) Raman spectra of pure powder of 4-MBT on Si substrate; Figure S6: SEM images of PSi films (a) before and (b) after transfer onto flexible tape coated with 40 nm Ag film; Figure S7: Raman spectra of R6G on the prepared SERS substrate at various CR6G ranging from 10^−7^ to 10^−12^ M; Figure S8: Raman spectra of the MPT residues on apple peels using flexible PSi films SERS substrate; Figure S9: SEM images of PSi films (a) before and (b) after 100 cycles of bending. (c) Raman spectra of 10^−4^ M 4-MBT collected on the flexible PSi films SERS substrate before and after 100 cycles of bending. References [45,46,47] are cited in the supplementary materials.
