# Supplementary material for: Wafer-Scale Fabrication and Transfer of Porous Silicon Films as Flexible Nanomaterials for Sensing Application
Source: Nanomaterials (Basel). 2022 Apr 2;12(7):1191. doi: 10.3390/nano12071191 (PMC9000722; doi:10.3390/nano12071191)
Supplement: Supplementary file 1 [file nanomaterials-12-01191-s001.zip › nanomaterials-1614290-supplementary.pdf]

nanomaterials

Supplementary Material

Wafer-Scale Fabrication and Transfer of Porous Silicon Films as Flexible Nanomaterials for Sensing Application

Han Lu [1], Mingliang Jin [2,3,*], Zongbao Zhang [4], Sujuan Wu [4] and Lingling Shui [1,2,3,*]

1 School of Information and Optoelectronic Science and Engineering, South China Normal University, Guangzhou 510006, China; hanlu@m.scnu.edu.cn
2 National Center for International Research on Green Optoelectronics & South China Academy of Advanced Optoelectronics, South China Normal University, Guangzhou 510006, China
3 International Academy of Optoelectronics at Zhaoqing, South China Normal University, Zhaoqing 526238, China
4 Institute for Advanced Materials, South China Academy of Advanced Optoelectronics, South China Normal University, Guangzhou 510006, China; zongbao.zhang@iapp.de (Z.Z.); sujwu@scnu.edu.cn (S.W.)
* Correspondence: jinml@scnu.edu.cn (M.J.); shuill@m.scnu.edu.cn (L.S.); Tel.: +86-20-3931-0508 (L.S.)

1. Schematic of PSi by Metal-Assisted Chemical Etching

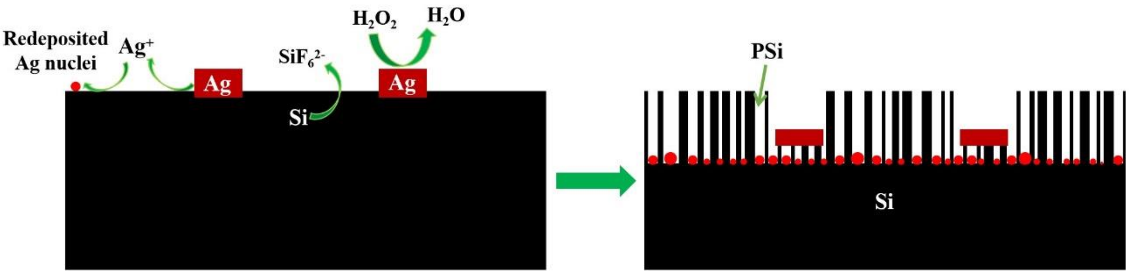

Figure S1. Schematic of PSi by metal-assisted chemical etching.

2. Uniformity of PSi Films

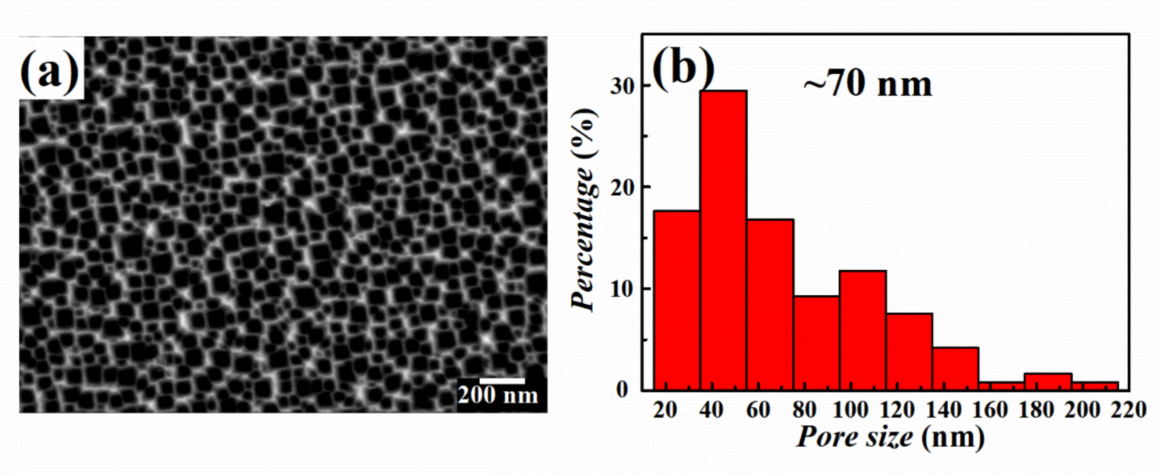

Figure S2. (**a**) SEM image of PSi films (a 65×65 μm^2 patterned substrate covered with ~120 nm Ag reaction in the etchant with R=6:4 for 30 min). (**b**) Corresponding distribution histogram of pore size.

3. Pore Size Distribution Histograms of PSi Films

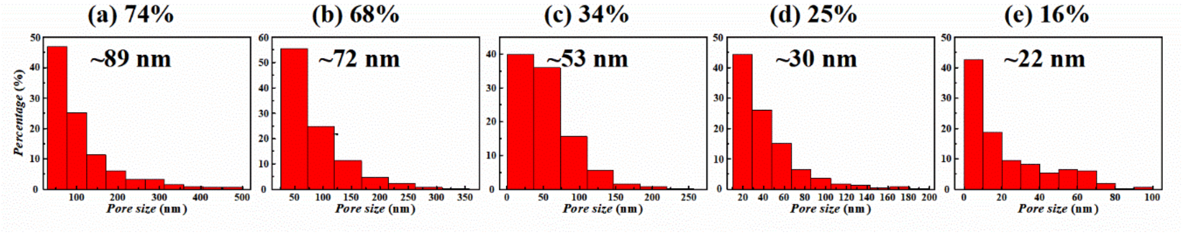

Figure S3. Corresponding pore size distribution histograms of PSi films with different porosity (**a**) 74%, (**b**) 68%, (**c**) 34%, (**d**) 25%, and (**e**) 16% (corresponding to Figures 2a–e).

4. Model of the Formation of Horizontal Cracks and Self-Peeling of PSi Films

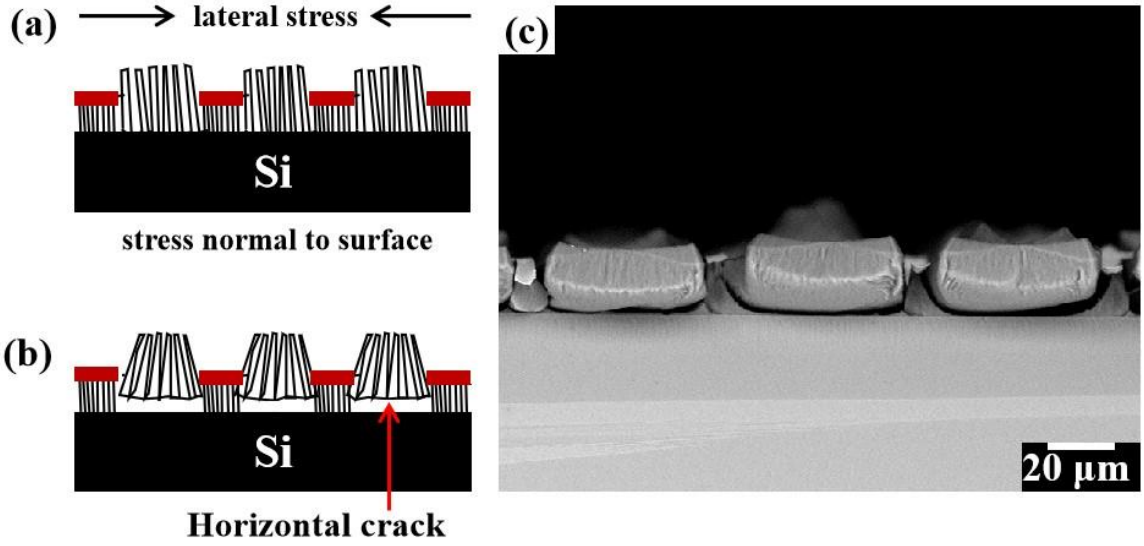

Figure S4. (**a**–**b**) Model of the formation of horizontal cracks of PSi films during drying. (**c**) SEM image of PSi films with horizontal cracks.

5. Calculation EF for 4-MBT

The experimental Raman EF of the SERS substrate was calculated by:

$$\mathrm{EF} = \frac{I_{SERS}}{I_{bulk}}\frac{N_{bulk}}{N_{SERS}} \tag{S1}$$

Where I_{SERS} denotes the Raman intensity of the 4-MBT on the Ag deposited PSi films SERS substrate and I_{bulk} represents the Raman intensity of the 4-MBT powder on Si substrate. N_{SERS} is the the number of 4-MBT molecules under the laser spot focus, and N_{bulk} is the number of the 4-MBT molecules contributed to the Raman spectra on pure powder, respectively.

N_{bulk} was determined by assuming that the laser excitation volume has a cylinder shape with the circular diameter being equal to the focused laser spot diameter and the height being equal to the effective probe depth (H_{obj}). H_{obj} was obtained by adjusting the substrate stage out of the laser focus plane in 1 μm increments and capturing the silicon characteristic peak value at 520 cm^{-1}. N_{bulk} is not counted when the signal intensity is less than half of the maximum value at the characteristic position, yielding the measured H_{obj} value is 26 μm. The amount of N_{bulk} that contributes to the pure powder Raman signal inside the interaction volume is calculated to be 1.76×10^{11} with the molar volume of 4-MBT 118.3 cm$^{3}\cdot$mol^{-1}.

MBT molecules are assumed to be absorbed as a monolayer with a surface density ($D_{\mathrm{4\text{-}MBT}}$) of 4.5×10^{18} molecules/m^2 onto the surface area [1]. The diameter of the laser spot in our Raman system is 1.30 μm. The area irradiated by the laser is 1.33×10^{-12} m^2. N_{SERS}

was obtained by multiplying the calculated total surface area (1.33 × 10^{-12} m^2) by the surface density of the 4-MBT molecules chemisorbed on the PSi surface ($D_{4\text{-MBT}}$ = 4.5 × 10^{18} molecules/m^2), yielding N_{SERS} = 5.98 × 10^6. The EFs of the SERS substrates (samples i-v in Figure 5a) were calculated to be ~1.12×10^7, ~2.85×10^7, ~1.68×10^7, ~0.56×10^7 and ~0.27×10^7 at the Raman peak of 1078 cm^{-1}, respectively.

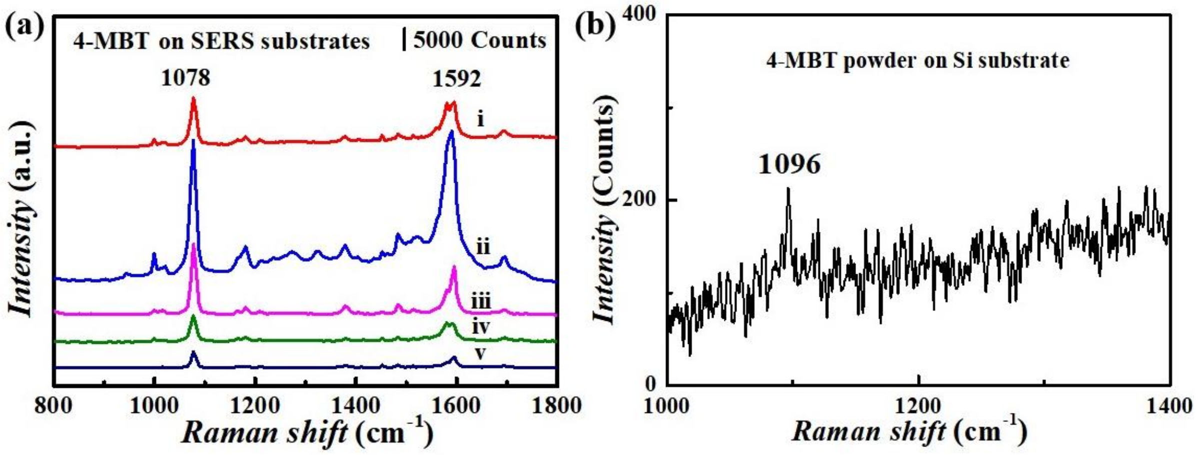

Figure S5. (**a**) Raman spectra of 10^{-4} M 4-MBT on PSi SERS substrates, (**b**) Raman spectra of pure powder of 4-MBT on Si substrate.

6. SEM Images of PSi Films Before and After Transfer to Tape Coated with Ag Film

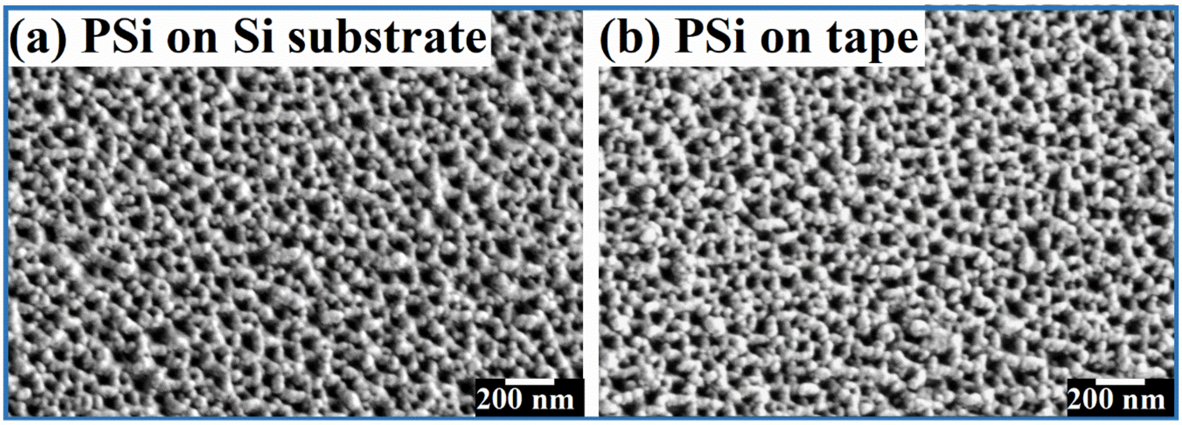

Figure S6. SEM images of PSi films (**a**) before and (**b**) after transfer onto flexible tape coated with 40 nm Ag film.

7. SERS Measurement of R6G

To further demonstrate the efficiency of the PSi SERS substrates, R6G with different molecular structure form 4-MBT has also been applied for SERS characterization. Figure S7 shows the Raman spectra of R6G on the as-prepared SERS substrate with C_{R6G} ranging from 10^{-7} to 10^{-12} M. At C_{R6G} = 10^{-12} M, the characteristic peaks of R6G were obvious. The detection limit was measured to be 10^{-12} M, demonstrating good SERS effect. The characteristic peaks of R6G were observed at 614, 771, 1187, 1362, 1507, 1571 and 1648 cm^{-1}. The peaks at 614, 771, and 1187 cm^{-1} are ascribed to C-C-C ring in-plane bending, C-H out-of-plane bending, and C-O-C stretching vibrations, respectively. And the peaks at 1362, 1507, 1571, and 1648 cm^{-1} are associated with aromatic C-C stretching vibrations [2].

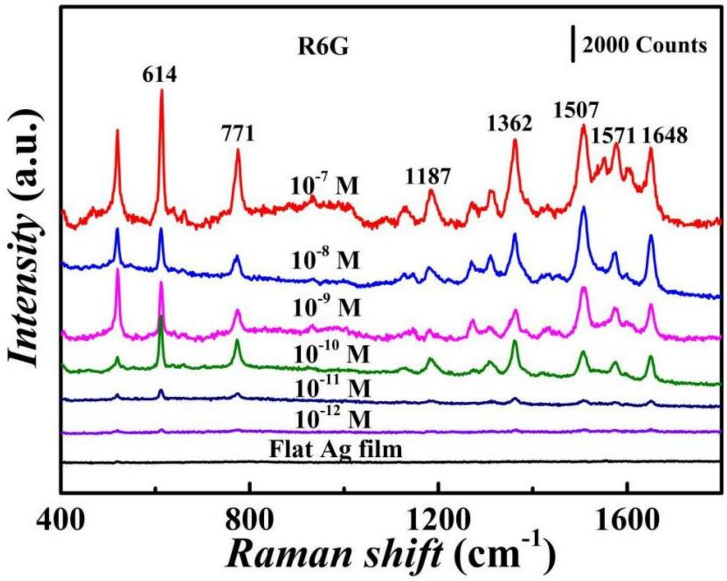

Figure S7. Raman spectra of R6G on the prepared SERS substrate at various C_{R6G} ranging from 10^{-7} to 10^{-12} M.

8. Detection of Pesticide Residues on Apple Peels

Due to excellent SERS performance, the flexible PSi films SERS substrates were used for detection of pesticide residues on apple surface with a real curved surface. Apple peels were cut into 1.0 × 1.0 cm^2 squares. Then, 10 μL of MPT solution with different concentrations was spread onto the peel surface and dried at room temperature, respectively. Afterward, 10 μL of ethanol was dropped onto the pretreated apple peel to extract the MPT. Subsequently, the flexible PSi SERS substrate was pressed to the apple peel for 10 s and peeled off slowly for further SERS analysis. The Raman spectra of MPT with C_{MPT} ranging from 10^{-4} to 10^{-6} M collected from the apple peels are shown in Figure S8. The characteristic peaks of MPT were observed at 849, 1152, and 1341 cm^{-1}. The peaks at 849 and 1152 cm^{-1} are ascribed to P-O stretching and C-N stretching vibrations, respectively. And the peaks at 1341 cm^{-1} are associated with aromatic C-H bend stretching vibrations [3]. The minimum detectable concentration of MPT was 10^{-6} M, converted to mass-to-area ratio was 2.6 × 10^{-9} g/cm^2, which is lower than the maximum residue limit for MPT in China and European Union [4].

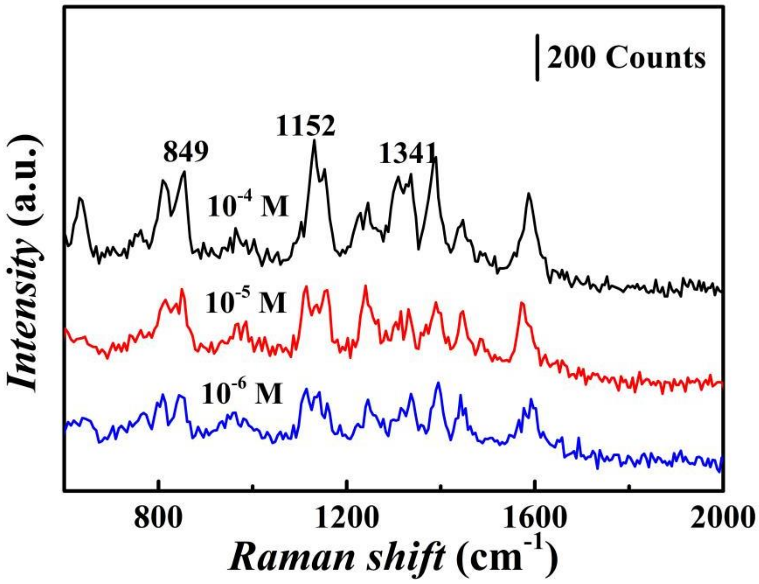

Figure S8. Raman spectra of the MPT residues on apple peels using flexible PSi films SERS substrate.

9. Bending Tests of Flexible PSi Films SERS Substrate

The bending test of the flexible PSi films SERS substrate was evaluated for the mechanically robust of SERS substrate for 100 cycles. For bending test, the flexible PSi films SERS substrates was bent in half. The SEM images and Raman spectra of flexible PSi films coated with 40 nm Ag film before and after 100 cycles of bending were presented in Figure S9, indicating that the flexible PSi films SERS substrate showed acceptable mechanical stability.

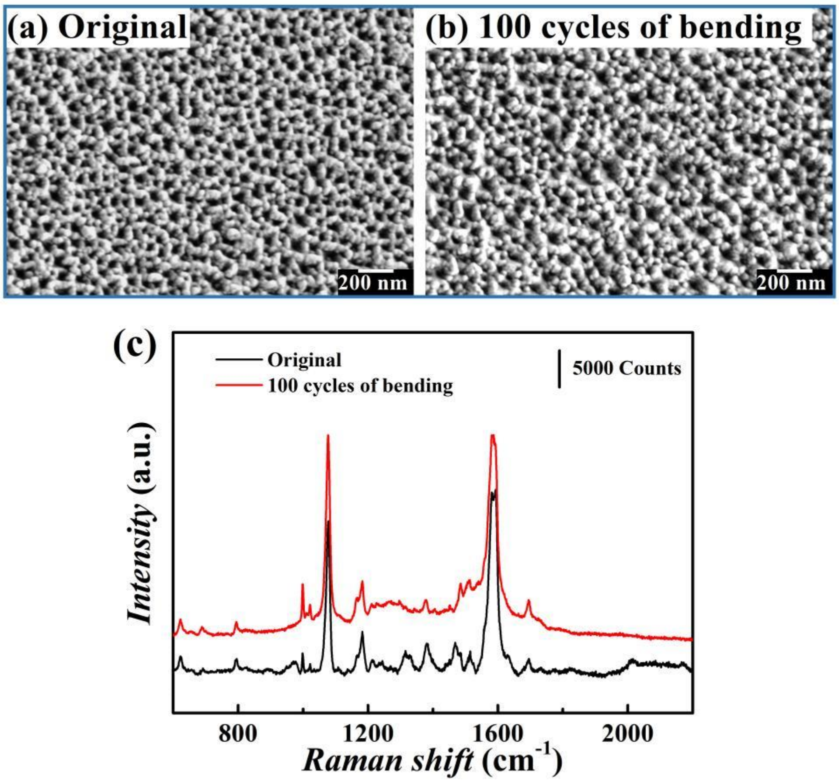

Figure S9. SEM images of flexible PSi films (**a**) before and (**b**) after 100 cycles of bending. (**c**) Raman spectra of 10^{-4} M 4-MBT collected on the flexible PSi films SERS substrate before and after 100 cycles of bending.

References

1. Lee, Y. H.; Shi, W.; Lee, H. K.; Jiang, R.; Phang, I. Y.; Cui, Y.; Isa, L.; Yang, Y.; Wang, J.; Li, S.; Ling, X. Y. Nanoscale surface chemistry directs the tunable assembly of silver octahedra into three two-dimensional plasmonic superlattices. *Nat. Commun.* **2015**, *6*, 6990, http://doi.org/10.1038/ncomms7990.
2. Jayrarn, N. D.; Aishwarya, D.; Sonia, S.; Mangalaraj, D.; Kumar, P. S.; Rao, G. M. Analysis on superhydrophobic silver decorated copper oxide nanostructured thin films for SERS studies. *J. Colloid Interf. Sci.* **2016**, *477*, 209–219, http://doi.org/10.1016/j.jcis.2016.05.051.
3. Wu, H.; Luo, Y.; Hou, C.; Huo, D.; Zhou, Y.; Zou, S.; Zhao, J.; Lei, Y. Flexible bipyra-mid-AuNPs based SERS tape sensing strategy for detecting methyl parathion on vegetable and fruit surface. *Sensor. Actuat. B-Chem.* **2019**, *285*, 123–128, http://doi.org/10.1016/j.snb.2019.01.038.
4. Wang, P.; Wu, L.; Lu, Z.; Li, Q.; Yin, W.; Ding, F.; Han, H. Gecko-inspired nanotentacle sur-face-enhanced Raman spectroscopy substrate for sampling and reliable detection of pesticide residues in fruits and vegetables. *Anal. Chem.* **2017**, *89*, 2424–2431, http://doi.org/10.1021/acs.analchem.6b04324.
